# Supplementary material for: The genetic profile of elite youth soccer players and its association with power and speed depends on maturity status
Source: PLoS One. 2020 Jun 22;15(6):e0234458. doi: 10.1371/journal.pone.0234458 (PMC7307776; doi:10.1371/journal.pone.0234458)
Supplement: Attachment — Submitted filename: Response to Reviewers.docx [file pone.0234458.s002.docx]

Dear Editor and Reviewers,

We are very grateful to you all for you taking the time to review our manuscript and for providing very constructive comments. We believe the changes we have made to our revised manuscript in response to these comments have significantly improved the quality of the manuscript, so thank you. We have considered all comments and responded to each one in turn.

Best wishes,

Rob Erskine on behalf of all co-authors

Editor Comments:

1. We note that you have included the phrase “data not shown” in your manuscript. Unfortunately, this does not meet our data sharing requirements. PLOS does not permit references to inaccessible data. We require that authors provide all relevant data within the paper, Supporting Information files, or in an acceptable, public repository. Please add a citation to support this phrase or upload the data that corresponds with these findings to a stable repository (such as Figshare or Dryad) and provide and URLs, DOIs, or accession numbers that may be used to access these data. Or, if the data are not a core part of the research being presented in your study, we ask that you remove the phrase that refers to these data.

AUTHORS’ RESPONSE*:* Thank you for raising this point. We feel that these data are important for providing further confidence in our main results. Therefore, we have included descriptions of the relevant physiological measurements in the Materials and Methods section (P 12-13), and the data in the results section (Table 3; P 24), as well as correlation data between the two TGSs and muscle physiological variables on P20 and P24:

*Results section, P24:*

**SNPs associated with skeletal muscle physiological variables**

In a sub-sample of 42 post-PHV participants (ESP, *n* = 22; and CON, *n* = 20), we found associations between three SNPs [*AGT* A>G (rs699), *COL5A1* C>T (rs12722) and *PPARA* G>C (rs4253778)] and quadriceps femoris muscle volume, PCSA, architecture and maximum strength (Table 3).

**Table 3.** **Quadriceps femoris muscle strength, size and architecture in 42 post-PHV soccer players (ESP, *n* = 22; and CON, *n* = 20).** Mean ± SD data are presented according to genotype for three different SNPs.

| **SNP** | **Quadriceps femoris muscle physiological property** | | | |
| --- | --- | --- | --- | --- |
|  | **KE MVC (N∙m)** | **QF *V*_m_ (L)** | **QF PCSA (cm^2^)** | **VL *θ*_p_ (°)** |
| ***AGT* A>G (rs699)** | | | | |
| TT (*n* = 6) | 252 ± 40 | 2.5 ± 0.2 | 203 ± 15 | 12.8 ± 1.3 |
| TC (*n* = 23) | 306 ± 61 | 2.5 ± 0.3 | 197 ± 31 | 14.9 ± 1.8 |
| CC (*n* = 13) | 349 ± 71** | 3.0 ± 0.6*** | 235 ± 48** | 15.3 ± 2.5* |
| ***COL5A1* C>T (rs12722)** | | | | |
| CC (*n* = 17) | 345 ± 70** | 2.8 ± 0.6* | 225 ± 43* | 14.8 ± 2.3 |
| CT (*n* = 18) | 297 ± 61 | 2.6 ± 0.3 | 207 ± 32 | 14.7 ± 2.1 |
| TT (*n* = 7) | 268 ± 46 | 2.4 ± 0.2 | 180 ± 26 | 14.6 ± 2.0 |
| ***PPARA* G>C (rs4253778)** | | | | |
| GG (*n* = 25) | 293 ± 61 | 2.6 ± 0.4 | 208 ± 34 | 14.4 ± 1.9 |
| GC (*n* = 13) | 343 ± 76 | 2.8 ± 0.5 | 204 ± 43 | 14.3 ± 1.9 |
| CC (*n* = 4) | 329 ± 59 | 2.9 ± 0.7 | 241 ± 47 | 17.9 ± 1.8*** |

*KE MVC*, knee extension maximum voluntary contraction; *QF V_m_*, quadriceps femoris muscle volume; *PCSA*, physiological cross-sectional area; *VL θ_p_*, vastus lateralis muscle fascicle pennation angle; *significantly greater than other genotypes for that respective SNP (*P* < 0.05); **significantly greater than other genotypes for that respective SNP (*P* < 0.02); ***significantly greater than other genotypes for that respective SNP (*P* < 0.01).

The ‘ESP’ TGS correlation data on P20:

… *“Regarding muscle physiological variables in post-PHV ESP and CON (n = 42), the ‘ESP’ TGS correlated with quadriceps femoris (QF) muscle volume (r = 0.400; P = 0.010), QF PCSA (r = 0.334; P = 0.033), VL θ_p_ (r = 0.416; P = 0.007) and knee extension MVC (r = 0.408; P = 0.008). In post-PHV ESP only (n = 22), the ‘ESP’ TGS correlated with QF muscle volume (r = 0.430; P = 0.046) and QF PCSA (r = 0.438; P = 0.041), VL θ_p_ (r = 0.441; P = 0.040) and knee extension MVC (r = 0.474; P = 0.026).”*

The ‘performance’ TGS correlation data on P24:

*Regarding muscle physiological variables in post-PHV ESP and CON (n = 42), the ‘performance’ TGS correlated with quadriceps femoris (QF) muscle volume (r = 0.422; P = 0.006), QF PCSA (r = 0.425; P = 0.006) and knee extension MVC (r = 0.388; P = 0.012). In post-PHV ESP only (n = 22), TGS correlated with QF muscle volume (r = 0.573; P = 0.005) and QF PCSA (r = 0.566; P = 0.006).*

2. Please, pay particular (but not limitedly to) attention to Reviewer 1's issues.

AUTHORS’ RESPONSE*:* Thank you. Once again, we are very grateful to have received such constructive comments from all three reviewers, and we have responded to all comments raised.

Reviewers' comments:

Reviewer's Responses to Questions

**Comments to the Author**

1. Is the manuscript technically sound, and do the data support the conclusions?
The manuscript must describe a technically sound piece of scientific research with data that supports the conclusions. Experiments must have been conducted rigorously, with appropriate controls, replication, and sample sizes. The conclusions must be drawn appropriately based on the data presented.
Reviewer #1: Yes

Reviewer #2: Yes

Reviewer #3: Yes

2. Has the statistical analysis been performed appropriately and rigorously?
Reviewer #1: No

AUTHORS’ RESPONSE*:* We have responded more comprehensively to Reviewer #1’s comments regarding our statistical approach in section 5, below. We believe our statistical analyses have been performed appropriately and extremely rigorously. This may not have been apparent in our original submission due to our desire to be succinct but, in response to Reviewer #1’s requests, we have included comprehensive information surrounding our statistical approaches in our revised manuscript, which should demonstrate the appropriateness and rigour of our statistical analyses.

Reviewer #2: Yes

Reviewer #3: Yes

3. Have the authors made all data underlying the findings in their manuscript fully available?

The [PLOS Data policy](https://hes32-ctp.trendmicro.com/wis/clicktime/v1/query?url=http%3a%2f%2fwww.plosone.org%2fstatic%2fpolicies.action%23sharing&umid=e8b73f30-3262-476c-8d09-86576e72a17d&auth=768f192bba830b801fed4f40fb360f4d1374fa7c-f6b9e023f2c27a8b8c36b1998fe058cb6b961ec0) requires authors to make all data underlying the findings described in their manuscript fully available without restriction, with rare exception (please refer to the Data Availability Statement in the manuscript PDF file). The data should be provided as part of the manuscript or its supporting information, or deposited to a public repository. For example, in addition to summary statistics, the data points behind means, medians and variance measures should be available. If there are restrictions on publicly sharing data—e.g. participant privacy or use of data from a third party—those must be specified.

Reviewer #1: Yes

Reviewer #2: Yes

Reviewer #3: Yes

4. Is the manuscript presented in an intelligible fashion and written in standard English?

PLOS ONE does not copyedit accepted manuscripts, so the language in submitted articles must be clear, correct, and unambiguous. Any typographical or grammatical errors should be corrected at revision, so please note any specific errors here.

Reviewer #1: Yes

Reviewer #2: Yes

Reviewer #3: Yes

5. Review Comments to the Author

Please use the space provided to explain your answers to the questions above. You may also include additional comments for the author, including concerns about dual publication, research ethics, or publication ethics. (Please upload your review as an attachment if it exceeds 20,000 characters)

Reviewer #1: The author studied the association of 10 SNPs and elite athlete status and power/speed performance in 536 youth soccer players and 151 age-matched controls. The main issue of this study is founding by population stratification. There are more Caucasians in the ESP group vs control group and many of the SNPs being investigated showed very different allele frequencies in Caucasian and African American population (e.g., MAF of rs4253778 in PPARA is 0.19 in Caucasian and 0.95 in African American). This will cause inflated Type I error rate (false positives). The analysis should either adjust for race or stratify on race. Without race information or a genomewide genotype data (to cluster the sample into different ethnic groups) the results are questionable.

AUTHORS’ RESPONSE: Thank you for raising this point. In the discussion of our originally submitted manuscript, we acknowledged that the inclusion of different ethnic groups may be regarded as a limitation of our study due to these points mentioned by Reviewer #1 (although the data we have seen from the 1000 genome project demonstrates a slightly smaller difference in rs4253778 MAF, e.g. 0.192 vs. 0.745 for EUR vs. AFR).

Although the absolute number of Caucasians was higher in ESP compared to CON (the overall group size of ESP was larger than CON, regardless of ethnic group), the relative proportions of Caucasians (and other ethnic groups) within ESP and the sub-sample of CON were similar. Thus, a comparison between two groups (ESP and CON) with similar relative ethnic group composition should enable a fair comparison. As the ANOVA model compares means between groups, it is unclear to us how our current analysis would have led to type I errors.

We accounted for the imbalance in the cohort sizes between ESP and CON by ensuring that all data were checked for violations of specific three-way ANOVA assumptions that may invalidate the interpretation of results. More specifically, Mauchly's test of sphericity, which assesses whether the variance of the differences between levels of the within-subject factors are equal, was applied to establish if the homogeneity of variance principle had been violated. If Mauchly's test of sphericity was violated and the variance of the differences between levels of the within-subject factors were not equal, the Greenhouse-Geisser correction method was applied [1]. This correction method therefore accounted for the imbalance in cohort sizes when analysing three-way ANOVAs. In addition, box plots were used to check if there were any outliers (none were found); and all data sets were tested for normality using the Kolmogorov-Smirnov test. Testing for such assumptions allowed us to be confident that the interpretation of the three-way ANOVAs was robust despite different sample sizes in CON and ESP.

We have added the following to the statistical analysis section of the manuscript on P16:

*We accounted for the imbalance in the cohort sizes between ESP and CON by ensuring that all data was checked for violations of specific three-way ANOVA assumptions that may invalidate the interpretation of results. More specifically, Mauchly's test of sphericity, which assesses whether the variance of the differences between levels of the within-subject factors are equal, was applied to establish if the homogeneity of variance principle had been violated. If Mauchly's test of sphericity was violated, the Greenhouse-Geisser correction method was applied* [1]*. To test for other assumptions, box plots were used to check if there were any outliers (none were found); and all data sets were tested for normality using the Kolmogorov-Smirnov test.*

Finally, as discussed in our originally submitted manuscript, elite soccer academies in the countries included in our study do not comprise athletes from solely one ethnic group (as demonstrated from our ethnicity data), and we felt it was important that our results were representative of actual soccer academies rather than one ethnic group. Thus, we aimed to recruit our CON group with a similar composition of ethnic groups to our ESP cohort. Further, if we were able to stratify according to ethnic group, our sample sizes (which are further broken down into different maturation and genotype groups), particularly for non-Caucasian participants, would become significantly smaller, thus reducing the statistical power of our study and increasing the risk of making type II errors, i.e. finding false negatives. This would also make the study design extremely complicated. Critically, however, we do not have ethnic information for all of our control group (as originally stated in Materials and Methods), so stratifying according to ethnic group with a CON group sample size of n=59 (which would be further broken down into different maturation and genotype groups) would reduce statistical power even further. Therefore, although the reviewer raises a valid point (which we have acknowledged in the Discussion), with respect, we maintain that including ethnic group as a co-variate in a three-way between groups ANCOVA model, or analysing our data separately, according to ethnic group, would undoubtedly lead to type II errors.

Table 2. Please double check all the statistical tests. Some do not seem to be right. For example, rs4253778 in PPARA, neither the comparison of 18.8% vs 25.5% nor 25.5% vs 18.9% had p-value <0.05. Some SNPs a dominant model was assumed but some were not. Is that purely based on an allele frequency cutoff?

AUTHORS’ RESPONSE: Thank you for raising this point. We had actually reported significant differences between the allele frequency distributions you have mentioned in our original submission – please see the footnote beneath the original table. In this case, the asterisk (*) represented a significant (P < 0.05) difference between post-PHV ESP and CON, while the ampersand symbol (&) represented a significant (P < 0.05) difference between post-PHV ESP and pre-PHV ESP. However, we apologise that this was not clear. We have replaced the ampersand symbol with a dagger symbol (✝) and highlighted the between group differences within each significant *χ*^2^ model (please see the newly inserted text in the Table 3 header). We hope this now removes any confusion.

The reason why a dominant model was assumed was indeed based on a low MAF, for which our threshold was 0.25 in Caucasian populations (as this group was the predominant ethnic group in our study). Thus, for any SNP with a MAF <0.25 in a Caucasian population, the relatively low number of participants with the minor allele homozygote genotype was combined with the heterozygotes for that SNP. The following information has now been added to the table header:

*“All SNPs were analysed using a co-dominant model (e.g. AA vs. Aa vs. aa) except when the minor allele frequency (MAF) for a Caucasian population* [2] *was <0.25, in which case a dominant model (e.g. AA vs Aa+aa) was used instead. P-values are presented for significant χ^2^ tests of homogeneity and between group differences are identified (P < 0.05), i.e. * difference between All ESP and CON; ** difference between post-PHV ESP and CON; *** difference between pre-PHV ESP and CON; ^✝^ difference between post- and pre-PHV ESP.”*

We thank Reviewer #1 for raising these points. The addition of this information certainly helps to clarify between group differences in allele/genotype frequency distribution and why we have pooled some genotypes but not others.

Table 2. When there are only two categories of a variable only one category needs to be shown in the table since the second categories carries identical information. For example, for C vs G alleles or CC/GG vs GG genotypes of PPARA rs4253778, only one allele and one genotype group need to be shown.

AUTHORS’ RESPONSE: We are unsure of what Reviewer #1 is referring to with this point, as C vs G is not the same as CC/GG vs GG (or even CC/CG vs GG). This can be demonstrated by visually comparing allele frequency with genotype frequency distribution within the same group for any given SNP. Allele frequency distribution was analysed separately to genotype frequency distribution for each SNP, hence why we reported both the allele and genotype frequency distributions, i.e. we did not compare allele frequency with genotype frequency, so we are unsure why we would present only one allele and one genotype group. The way we have chosen to present these data is a common method of reporting allele and genotype frequency distributions for multiple SNPs.

Table 2. Please show actual p-values. Please incorporate rs number in the table
The authors mentioned FDR to correct for multiple testing but I don’t find FDR results in the paper. It is unclear how FDR were used since there are many statistical tests in this paper, over all p-values?

It is mentioned that “three-way between-subjects ANOVA was used to investigate the association between genotype…”. This is will unbalance design. It is not clear what terms were included in the anova, two-way interaction? three-way interaction?

Page 17, line 295, please double check the p-value.

AUTHORS’ RESPONSE: Thank you for raising these points. The reason we did not originally include P values in the table was to avoid including too much information, which may confuse the reader. However, we have now reformatted the table to accommodate the reviewer’s request and we agree that the addition of P values to the table is more informative.

The rs numbers were originally reported in the table heading but we have moved these to the appropriate places within the actual table, as the reviewer has requested. We agree that this avoids duplication of unnecessary information and makes the table clearer.

An earlier draft of this manuscript included more detail surrounding our FDR methods (both for our *χ*^2^ tests of homogeneity and three-way between subjects ANOVAs) but we decided to remove this in an attempt to make the manuscript more concise. However, we agree that the addition of this information would make the manuscript more informative, so we have now re-instated the relevant information in the Statistics section on P15. This now explains the FDR for the *χ*^2^ tests of homogeneity, the 3-way ANOVAs (associations between SNP and performance phenotypes) and the one-way ANOVAs for the newly introduced SNP associations with quadriceps femoris muscle physiological characteristics. The FDR thresholds for each SNP P value were calculated according to Benjamini and Hochberg [3].

Regarding the three-way ANOVA interactions and main effects investigated in this study, we focussed on the three-way interaction (genotype x athlete status x maturation), two-way interaction (genotype x athlete status and genotype x maturation status), and main effect of genotype. If a significant three-way interaction occurred, simple simple main effects and simple simple pairwise comparisons with Bonferroni adjustment revealed significant differences. If a significant two-way interaction occurred, simple main effects and pairwise comparisons with Bonferroni adjustment were performed to reveal differences. If a significant genotype main effect existed, Bonferroni post-hoc tests were used to locate the genotype difference in test performance scores. We have included the following in P16:

*Thus, we focussed on the three-way interaction (genotype x athlete status x maturation), two-way interactions (genotype x athlete status and genotype x maturation status), and main effect of genotype. If a significant three-way interaction occurred, simple simple main effects and simple simple pairwise comparisons with Bonferroni adjustment revealed significant differences. If a significant two-way interaction occurred, simple main effects and pairwise comparisons with Bonferroni adjustment were performed to reveal differences. If a significant genotype main effect existed, Bonferroni post-hoc tests were used to locate the genotype difference in test performance scores.*

Furthermore regarding the three-way ANOVAs, we accounted for the imbalance in the cohort sizes between ESP and CON by ensuring that all data was checked for violations of specific three-way ANOVA assumptions that may invalidate the interpretation of results. More specifically, Mauchly's test of sphericity, which assesses whether the variance of the differences between levels of the within-subject factors are equal, was applied to establish if the homogeneity of variance principle had been violated. If Mauchly's test of sphericity was violated and the variance of the differences between levels of the within-subject factors were not equal, the Greenhouse-Geisser correction method was applied [1]. This correction method therefore accounted for the imbalance in cohort sizes when analysing three-way ANOVAs. Box plots were used to check for outliers (none were found), and data sets were normally distributed according to the Kolmogorov-Smirnov test. Testing for such assumptions allowed us to be confident that the interpretation of the three-way ANOVA was robust despite different sample sizes in each group.

We have added the following to the statistical analysis section of the manuscript on P16:

*We accounted for the larger cohort size in ESP vs. CON by ensuring that all data was checked for violations of specific three-way ANOVA assumptions that may invalidate the interpretation of results. More specifically, Mauchly's test of sphericity, which assesses whether the variance of the differences between levels of the within-subject factors are equal, was applied to establish if the homogeneity of variance principle had been violated. If Mauchly's test of sphericity was violated, the Greenhouse-Geisser correction method was applied* [4]*. Box plots were used to check for outliers (none were found); and all data sets were normally distributed according to the Kolmogorov-Smirnov test.*

Finally, thank you for pointing out the typo in the P-value concerning lack of difference between pre- and post-PHV ESP in ‘performance’ TGS. This P-value was actually 0.227, not 0.022, which now agrees with the statement that there was no difference between pre-PHV ESP and CON (*P* = 0.227). We have also highlighted the lack of difference between pre-PHV ESP and CON (*P* = 0.609).

Once again, we are grateful to Reviewer #1 for raising all of these important points, and we feel that the changes we have made as a consequence have improved the quality of the manuscript.

Reviewer #2: The study is well designed and presented. My only caution are the statements suggesting that the genetic information could be used for talent identification purposes. This is a controversial issue as a result of the claims by may DTC companies. I suggest that the authors focus on the genetic contribution to the studied phenotype.

AUTHORS’ RESPONSE: Thank you for raising this important point. As this is the first study of its kind to investigate the genetic association with power and speed in elite youth soccer players (accounting for the potentially confounding factor of maturation), we wanted to highlight that our results provide preliminary evidence for genetic analyses being a potentially useful tool for **future** talent ID and development (i.e. after our results have been replicated by independent groups), and only in conjunction with traditional methods for talent ID and development. However, we completely agree with the reviewer’s concern about DTCs currently making false claims, e.g. that we are already at the point where genetic testing can be used for talent ID, injury risk, etc. Hence, our use of the words “assist” and “future” in our original concluding sentence to emphasise this point. On reflection and following the reviewer’s comment, we think this could still be misused by DTC companies as ‘current evidence’ that validates their ‘product’. Therefore, to prevent our findings being mis-represented by DTC companies, we have changed our concluding statement to the following:

*“Elite soccer academies may wish to consider this information when recruiting talented pre-PHV players, with a view of developing those players for the different demands of the post-PHV game, rather than relying on the recruitment of new players at that stage of maturation. Furthermore, before genetic testing can be considered a potentially useful tool to assist in talent identification and development, replication of our results by independent groups and the identification of more genetic markers of performance is necessary.”*

We have also revised our concluding statement in the abstract accordingly:

*“To conclude, we have shown for the first time that pre- and post-PHV ESP have distinct genetic profiles, with pre-PHV ESP more suited for endurance, and post-PHV ESP for power and speed (the latter phenotypes being crucial attributes for post-PHV ESP). We have also demonstrated that power, acceleration and sprint performance were associated with five SNPs, both individually and in combination, possibly by influencing muscle size and neuromuscular activation.”*

We hope Reviewer #2 agrees that our take home message has been satisfactorily changed to ensure it is unequivocal in terms of our study not supporting the false claims made by DTC companies.

Reviewer #3:

1. In the abstract, please confirm that the subjects were all male.

AUTHORS’ RESPONSE: Thank you for highlighting this omission. We have now included the word “male” in the Abstract.

2. INTRODUCTION:
   • In the context of athlete’s genetic studies more comprehensive review of literature is required.

AUTHORS’ RESPONSE: Thank you for raising this point. As the reviewer is aware, the challenge with writing a good introduction is to include a comprehensive review of the literature to provide a solid background to the study, as well as a convincing rationale for the study, all as succinctly as possible. To do this, we decided to focus on the literature pertaining to the genetic association with elite soccer player status/performance. We have also attempted to review the current literature when introducing the SNPs of interest in the Introduction, e.g. regarding the (potential) mechanistic influence of each SNP. However, we agree with Reviewer #3’s comment that more comprehensive information could be provided regarding the SNPs investigated in the current study. Therefore, in response to this and Reviewer #3’s subsequent comment, we have included a *NEW* Table 2, which provides functional information on each of the 10 SNPs (plus their respective genes and proteins), supported by both classic and recent literature. We have also added the following part of a sentence and reference pertaining to a recent review of the genetic association with power and speed phenotypes [5] on P3: *“Over 69 genetic markers have been associated with power athlete status* [5]*”* and the genetic association with power and speed is likely due to specific gene variants influencing protein abundance/expression, which will in turn affect the physiological determinants of speed and power.

• There is a lack of information about the function of 10 genes and their polymorphisms. Given that this is the premise of the research, this should be included. For example, a table could be provided describing the functions of genes and SNPs.

AUTHORS’ RESPONSE: Thank you for raising this point. In our originally submitted manuscript, we introduced each of our investigated SNPs in the Introduction but we agree that more information about the individual SNPs would be beneficial. Consequently, and in response to the reviewer’s excellent suggestion, we have added this information in our *NEW* Table 2 on P7. In this table, we have included the gene name, the protein product, the alleles and rs number, location, MAF, and SNP type and function (supported by both classic and recent literature). We feel this provides significantly more information about each SNP, so thank you for raising this.

3. What do the authors mean with " talent identification and development programmes " ? This point should be clarified.

AUTHORS’ RESPONSE: Thank you for raising this point. Talent identification refers to the process of recognising and recruiting individuals with the perceived potential to become elite players. Talent development is a process whereby the player is provided with the optimal learning environment to achieve their full potential. Consequently, a talent development programme is the intervention programme prescribed to provide the most optimal environment to allow the player to realise their full potential [6,7]. We have now clarified “talent identification and development programmes” in the Discussion on P25:

*The combination of these five SNPs explained a proportion of the variability in all performance tasks in pre-PHV ESP, but only the variability in bilateral horizontal-forward countermovement jump (BH CMJ) performance in post-PHV ESP. Together, these genetic differences suggest the soccer talent identification procedures (i.e. the process of recognising and recruiting individuals with the perceived potential to become elite players* [6,7]*) employed by the multiple academies in this study recruit pre- and post-PHV ESP based on different physiological criteria, i.e. endurance (pre-PHV) vs. power/speed (post-PHV) capability. Given the importance of power/speed in determining post-PHV ESP status* [8–12]*, these findings have significant implications for future youth soccer talent identification and development programmes (i.e. intervention programmes prescribed to provide the most optimal environment to allow the player to realise their full potential* [6,7]*).*

4. In the Results section, lines 256 and 280, please check the numbering of the tables.

AUTHORS’ RESPONSE: Thank you for pointing this out. We noticed that in some instances the *COL2A1* and *COL5A1* figure references were mixed up and we have now corrected the as *COL2A1* is Figure 2 and *COL5A1* is figure 3. We have also removed the asterisk from Figure 4 (*NOS3* association with 20 m sprint), as this was a non-significant tendency for a main effect.

6. PLOS authors have the option to publish the peer review history of their article ([what does this mean?](https://hes32-ctp.trendmicro.com/wis/clicktime/v1/query?url=https%3a%2f%2fjournals.plos.org%2fplosone%2fs%2feditorial%2dand%2dpeer%2dreview%2dprocess%23loc%2dpeer%2dreview%2dhistory&umid=e8b73f30-3262-476c-8d09-86576e72a17d&auth=768f192bba830b801fed4f40fb360f4d1374fa7c-663f5d432ec9d4bcb2bdd3f863b07fe3e272a34e)). If published, this will include your full peer review and any attached files.

If you choose “no”, your identity will remain anonymous but your review may still be made public.

**Do you want your identity to be public for this peer review?** For information about this choice, including consent withdrawal, please see our [Privacy Policy](https://hes32-ctp.trendmicro.com/wis/clicktime/v1/query?url=https%3a%2f%2fwww.plos.org%2fprivacy%2dpolicy&umid=e8b73f30-3262-476c-8d09-86576e72a17d&auth=768f192bba830b801fed4f40fb360f4d1374fa7c-644a97e3d61e2d6d11810f5b4fb9f8227c2d69d9).

Reviewer #1: No

Reviewer #2: Yes: Malcolm Collins

Reviewer #3: No

**References**

1.	Maxwell SE, Delaney HD, Kelley K. Designing experiments and analyzing data: A model comparison perspective. 3^rd^ ed. New York: Routledge; 2018.

2.	Auton A, Abecasis GR, Altshuler DM, Durbin RM, Abecasis GR, Bentley DR, et al. A global reference for human genetic variation. Nature. 2015;526: 68–74. doi:10.1038/nature15393

3.	Benjamini Y, Hochberg Y. Controlling the false discovery rate: a practical and powerful approach to multiple testing. J R Stat Soc Ser B. 1995; 289–300.

4.	Maxwell SE, Delaney HD. Designing experiments and analyzing. Lawrence Erlbaum Associates, Hillsdale. Chapters; 2004.

5.	Maciejewska-Skrendo A, Cięszczyk P, Chycki J, Sawczuk M, Smółka W. Genetic Markers Associated with Power Athlete Status. J Hum Kinet. 2019;68: 17–36. doi:10.2478/hukin-2019-0053

6.	Williams AM, Reilly T. Talent identification and development in soccer. J Sports Sci. 2000;18: 657–667.

7.	Sarmento H, Anguera MT, Pereira A, Araujo D. Talent identification and development in male football: a systematic review. Sport Med. 2018;48: 907–931.

8.	Murtagh CF, Brownlee TE, O’Boyle A, Morgans R, Drust B, Erskine RM. Importance of Speed and Power in Elite Youth Soccer Depends on Maturation Status. J strength Cond Res. 2018;32: 297–303. doi:10.1519/JSC.0000000000002367

9.	Murtagh CF, Stubbs M, Vanrenterghem J, O’Boyle A, Morgans R, Drust B, et al. Patellar tendon properties distinguish elite from non-elite soccer players and are related to peak horizontal but not vertical power. Eur J Appl Physiol. 2018;118: 1737–1749. doi:10.1007/s00421-018-3905-0

10.	Murtagh CF, Nulty C, Vanrenterghem J, O’Boyle A, Morgans R, Drust B, et al. The Neuromuscular Determinants of Unilateral Jump Performance in Soccer Players Are Direction-Specific. Int J Sports Physiol Perform. 2018;13: 604–611. doi:10.1123/ijspp.2017-0589

11.	Murtagh CF, Naughton RJ, McRobert AP, O’Boyle A, Morgans R, Drust B, et al. A Coding System to Quantify Powerful Actions in Soccer Match Play: A Pilot Study. Res Q Exerc Sport. 2019; 1–10.

12.	Murtagh CF, Vanrenterghem J, O’Boyle A, Morgans R, Drust B, Erskine RM. Unilateral jumps in different directions: a novel assessment of soccer-associated power? J Sci Med Sport. 2017;20: 1018–1023. doi:10.1016/j.jsams.2017.03.016
