# Supplementary material for: The genetic profile of elite youth soccer players and its association with power and speed depends on maturity status
Source: PLoS One. 2020 Jun 22;15(6):e0234458. doi: 10.1371/journal.pone.0234458 (PMC7307776; doi:10.1371/journal.pone.0234458)
Supplement: Attachment — Submitted filename: Response to Reviewers.docx [file pone.0234458.s003.docx]

Dear Editor and Reviewers,

Once again, we are very grateful to you all for taking the time to review our revised manuscript and for providing feedback for a second time, so thank you.

Following your initial review of our original manuscript (submitted to PLoS ONE on 27^th^ September 2019), we made significant changes in response to your welcomed comments, and we felt that we addressed all of them in a very comprehensive and fair manner.

We are very pleased that Reviewer #2 and Reviewer #3 have confirmed their satisfaction with our changes and that they feel that our manuscript has scientific value and is suitable for publication. We have responded to Reviewer #1’s additional comments, below.

As our manuscript has been under review for seven months at the time of submitting our second revised manuscript, we would greatly appreciate it if this review could be expedited. Thank you for your understanding.

Best wishes,

Rob Erskine, on behalf of all co-authors

Reviewer #1:

1. Population stratification is still a big concern in this revision as commented before. The proportions of white vs black is not as similar as stated by the authors in the responses. In the paper it says “all ESP (white, 79%; black, 5%; black/white, 16%; and Asian, 1%) and a sub-sample (n = 59) of CON (white, 93%; black, 2%;mixed black/white, 3%; and Asian, 2%).” To me, 79% is pretty difference from 93%. Given that the majority of the samples are white, at least a subset analysis using only white is warranted.

AUTHORS’ RESPONSE*:* As we stated in our original response to Reviewer #1’s comment on population stratification, we only have ethnic origin data in 38% of the control group. Therefore, we felt that making a comparison between Caucasian only ESP (n=422) and Caucasian only CON (n=54) would be unhelpful due to significantly reduced statistical power and the inevitability of making type II errors. Given the similarities between ESP and CON in terms of anthropometry, age and maturity status, and the similar percentages of various ethnic groups within ESP and the sub-sample of CON, we maintain that our genetic comparisons are still valid and our results compelling. However, in response to Reviewer #1’s request, we have investigated genotype/allele frequency distribution in participants of known Caucasian origin (ESP, *n* = 422; CON, *n* = 54), and found that *ACTN3* XX genotype frequency distribution was higher (*P* = 0.004) in pre-PHV ESP (32.0%) compared to both CON (13.0%) and post-PHV ESP (20.4%), which supports our total cohort data.

Consequently, we have included the following in the Discussion on P33 of the revised manuscript:

*“Nevertheless, despite ethnicity information only being available in 38% of CON, sub-sample analysis in participants of known Caucasian origin (ESP, n = 422; CON, n = 54) revealed that ACTN3 XX genotype frequency distribution was higher (P = 0.004) in pre-PHV ESP (32.0%) compared to both CON (13.0%) and post-PHV ESP (20.4%). This therefore supports our total cohort data.”*

2. Many hypotheses were tested in this paper, 10 SNPs x 5 outcome variables x 2 comparisons =100 already even before considering the two-way, three-way interactions. Adjusting for 10 tests is not enough. In addition, it is better to focus the paper on a smaller number of hypothesis. The sample size is limited in detection interaction effects, especially the three-way interaction.

AUTHORS’ RESPONSE*:* Each ANOVA model accounts for the multiple P values derived from the main effects and interaction effects within that model, i.e. main effect of genotype, 2-way interactions between genotype and maturation, 2-way interaction between genotype and athlete group, and three-way interaction between genotype, maturation and athlete group.

It is also perfectly reasonable to perform a three-way ANOVA for each dependent variable (vertical CMJ, horizontal CMJ, agility, acceleration and sprint), as each of these variables are assessments of different aspects of physical performance. Thus, it is reasonable to hypothesise that some SNPs may be associated with just one or several of these performance variables, depending on the function of the SNP(s), which is not known in all of our 10 SNPs.

We investigated the association 10 SNPs with these five performance variables. Therefore, we have performed 10 multiple comparisons within the same data set. This is why each of our FDR models (for the main effect and each of the interaction effects) included 10 P values. We feel that we have satisfactorily addressed this in our response to Reviewer #1’s initial comment regarding our statistical approach, i.e. as requested, we detailed our robust and rigorous statistical approach in our revised manuscript.

3. Total genotype score based on SNPs that demonstrating differences is biased. Creating scores using genotypes from all 10 SNPs is not susceptible to such bias and it also reduces the number of comparisons.

AUTHORS’ RESPONSE*:* We respectfully disagree with this comment. Firstly, the ‘performance’ TGS was calculated on the basis of a SNP being associated with any performance variable, and it could be associated solely with ESP or CON, or a particular maturation group within one or both groups. Therefore, we do not see how this TGS could be biased. Secondly, the ‘ESP’ TGS includes SNPs that had a higher/lower genotype/allele frequency distribution in one or multiple maturation groups in ESP vs. CON. We included only the associated SNPs, because they were the only ones of interest (we did not see any scientific justification for increasing the noise by including non-associated SNPs). Further, if a SNP is not associated with a dependent variable, we cannot be confident which genotype/allele is advantageous/disadvantageous. Referring to previous literature for this is not a viable solution, because ours is a completely novel population. Moreover, some of these SNPs have not been investigated in a sporting context before, making it impossible to know which is the advantageous genotype/allele. Finally, just because one SNP has a higher/lower genotype/allele frequency distribution does not mean that, when you include other SNPs in a combined TGS model, that the TGS will be higher in one cohort vs. another. This is because all ESP participants may not have all five beneficial genotypes. Indeed they did not but what was interesting (and the reason we performed the analysis this way) was that the ‘ESP’ TGS was higher in ESP than in CON, therefore providing evidence that a larger proportion of ESP had more of the *beneficial* genotypes than CON. Moreover (and another reason our TGS models were not biased), not all SNPs were associated with the same maturation group in ESP or CON but the TGS comparisons were total ESP TGS *vs.* total CON TGS.

4. The comment in the previous review: “Table 2. When there are only two categories of a variable only one category needs to be shown in the table since the second categories carries identical information. For example, for C vs G alleles or CC/GG vs GG genotypes of PPARA rs4253778, only one allele and one genotype group need to be shown.” What I meant is that if frequency of C allele is listed frequency of the G allele is just one minus that. But it is fine to leave both in. I would recommend putting actual counts in addition to frequencies in the table as well.

AUTHORS’ RESPONSE*:* We feel that including counts as well as percentages would clutter an already busy table. The sample sizes for each group are stated at the top of the table, and readers can calculate actual counts themselves using the percentages, if necessary.

Reviewer #2:

The revised manuscript is improved since the authors have addressed all the comments and concerns raised by the reviewers.

AUTHORS’ RESPONSE*:* Thank you for confirming your satisfaction with our revised manuscript. We are very grateful to you for your time and feedback, which has helped improve our manuscript.

Reviewer #3:

The authors have made good efforts to address the issues raised. The manuscript has scientific value and is suitable for publication.

AUTHORS’ RESPONSE*:* Thank you also for confirming your satisfaction with the extensive changes we made to our manuscript following the first revision. We are very grateful for your time and feedback too, and we have no doubt that this has helped improve our manuscript.
