# Supplementary material for: The genetic profile of elite youth soccer players and its association with power and speed depends on maturity status
Source: PLoS One. 2020 Jun 22;15(6):e0234458. doi: 10.1371/journal.pone.0234458 (PMC7307776; doi:10.1371/journal.pone.0234458)
Supplement: Attachment — Submitted filename: Response to Reviewers.docx [file pone.0234458.s004.docx]

Dear Editor,

We have responded to Reviewer #1’s additional comments, below.

As our manuscript will have been under review for over seven months at the time of submitting the fourth version of our manuscript, we would greatly appreciate it if this final review could be expedited. Thank you for your understanding.

Best wishes,

Rob Erskine, on behalf of all co-authors

Reviewer #1:

1. Regarding response to #1, not trying to be picky on words, however performing tests using Caucasians only does not “inevitably” introduce type II error since for type II error to occur the alternative hypothesis has to be true and we do not know if that is the case. Only result of ACTN3 XX genotype is mentioned what about the other SNPs? It will be reassuring if the results of the Caucasian-analyses show similar trend, though not necessarily reaching statistical significance.

AUTHORS’ RESPONSE*:* If the sample size is small, it increases the chance of making type II errors. Only using 38% of the CON group (not because they were the only Caucasian CON participants but because they were the only ones we had ethnicity information for) severely reduces the sample size, and therefore increases the chance of making type II errors. We were willing to report the *ACTN3* genotype frequency data in Caucasian only participants in response to Reviewer #1’s request, because it did not confuse the main message of the manuscript. Due to only including 38% CON, the other genotype/allele data showed a similar trend to the overall genotype/allele data but did not reach statistical significance (*PPARA* GG: post-PHV ESP 59.2%, pre-PHV ESP 68.9%, CON 72.2%, *P* = 0.069; *PPARA* G-allele: post-PHV ESP 77.2%, pre-PHV ESP 83.0%, CON 85.2%, *P* = 0.060; *AGT* AA: post-PHV ESP 24.6%, pre-PHV ESP 35.0%, CON 27.8%, *P* = 0.133; *AGT* A-allele: post-PHV ESP 49.3%, pre-PHV ESP 56.8%, CON 56.5%, *P* = 0.109; and *NOS3* T-allele: ESP 63.5%, CON 57.4%, *P* = 0.217). However, including those under-powered data would confuse the manuscript considerably but we trust this similar trend satisfies the reviewer. We believe including the Caucasian only *ACTN3* genotype frequency information in the revised manuscript is a fair compromise.

2. Regarding correction for multiple testing, I maintain my point of view that too many tests have been performed and the number of tests to be adjusted for should be more than 10. Having said that, the FDR adjusted p-values should be reported along with the unadjusted p-values so that the readers can weigh the evidence themselves. On line 247-248 “To control for multiple comparisons (10 SNPs and five performance variables), ….” , “and five performance variables” should be removed since they were not being controlled by the current FDR adjustment. Also a better place for this sentence is on line 241 after “main effects of genotypes”. This way it describes how overall association is evaluated before describing post-hoc comparison.

AUTHORS’ RESPONSE*:* As explained in our initial *Response to reviewers’ comments* document, and in the first revised manuscript, the original (“unadjusted”) P values were compared with the respective B-H critical values (line 225-229). Reporting “adjusted” B-H P values can be confusing in itself but adding this information to the original (“unadjusted”) P values would complicate and confuse the results section, and the overall manuscript. Comparing the original (unadjusted) P values with their respective B-H critical values is normal practice and we see no reason to change this. However, as per Reviewer #1’s request. we have removed “and five variables” from line 247 and moved this sentence to line 241.

3. Regarding bias in “TGS”, even though the SNPs were selected based on different performance outcomes it is conceivable that these outcomes are also associated with ESP and CON group status. Therefore this preselection process can be biased.

AUTHORS’ RESPONSE: For a full rebuttal to this comment, please refer to our previous *Response to reviewers’ comments* document. In brief, the ‘performance’ TGS comprised SNPs that were associated with different performance variables in CON and/or ESP at different stages of maturity. Therefore, we maintain there was no bias.
